# Neutrophil phenotypes quantify tissue damage caused by major surgery

**DOI:** 10.3389/fsurg.2025.1494831

**Published:** 2025-03-07

**Authors:** Emma J. de Fraiture, Ted Reniers, Nathalie E. W. Vreeman, Thijs C. D. Rettig, Hjalmar C. van Santvoort, Angela Bikker, Nienke Vrisekoop, Leo Koenderman, Falco Hietbrink, Peter G. Noordzij

**Affiliations:** ^1^ Department of Trauma Surgery, University Medical Center Utrecht, Utrecht, Netherlands; ^2^ Department of Anesthesiology, Intensive Care and Pain Medicine, St. Antonius Hospital, Nieuwegein, Netherlands; ^3^ Department of Intensive Care, University Medical Center Utrecht, Utrecht, Netherlands; ^4^ Department of Anesthesiology, Intensive Care and Pain Medicine, Amphia Hospital, Breda, Netherlands; ^5^ Department of Surgery, Regional Academic Cancer Center Utrecht, St Antonius Hospital Nieuwegein, Utrecht, Netherlands; ^6^ Department of Clinical Chemistry, St. Antonius Hospital, Nieuwegein, Netherlands; ^7^ Department of Respiratory Medicine, University Medical Center Utrecht, Utrecht, Netherlands; ^8^ Center for Translational Immunology, University Medical Center Utrecht, Utrecht, Netherlands

**Keywords:** inflammation, immune response, surgery, CABG, surgery for pancreatic cancer, tissue damage

## Abstract

**Introduction:**

Major surgery triggers an innate immune response that can become excessive, leading to immune suppression and an increased risk of infection. Neutrophils are crucial in this response, and changes in their phenotype are associated with the severity of the innate immune response. This study examines the effect of major surgery on neutrophil phenotypes using fully automated flow cytometry.

**Methods:**

In this prospective single-center cohort study, adult patients undergoing either pancreaticoduodenectomy or on-pump coronary artery bypass grafting (CABG) were enrolled in the (BIGPROMISE) study. Blood samples were collected preoperatively (after anesthesia induction) and postoperatively (immediately after surgery). Neutrophil phenotypes were assessed using automated flow cytometry, with a rapid analysis time of less than 30 min.

**Results:**

The study included 24 patients undergoing CABG and 12 patients undergoing pancreaticoduodenectomy. Preoperative neutrophil heterogeneity was minimal, but significant postoperative changes in neutrophil subsets were observed in all patients, indicating acute systemic inflammation. Patients who underwent pancreatic surgery showed a more extensive inflammatory response, with 83% in Category 5, compared with 29% in the CABG group.

**Conclusions:**

This is the first study to use fully automated flow cytometry to monitor perioperative changes in neutrophil phenotypes following major surgery. Our findings provide an in-depth readout of the innate immune response and neutrophil activation, highlighting a more pronounced response to pancreatic surgery than to cardiac surgery. Neutrophil phenotyping could serve as a valuable biomarker for patient stratification and management, although larger cohort studies are needed to confirm its predictive value for postoperative complications.

## 1 Introduction

Major surgery triggers an innate immune response and systemic inflammation, which is normally self-limiting (1). However, the postoperative immune response can lead to excessive, prolonged, and aberrant (systemic) inflammation, which is associated with immune suppression and, thereby, an increased risk of infection (2). The immune response to tissue damage is initiated at the moment of (surgical) injury, triggering parallel processes, namely, a proinflammatory response that increases the risk of organ failure and immune paralysis that heightens susceptibility to infections (3). Neutrophils play a key role in this inflammatory-immune response to tissue injury (4). In patients with major trauma, changes in neutrophil phenotypes have been associated with injury severity and infectious complications and as such, may serve as a read-out of the innate immune response during and after major elective surgery (5, 6).

A bedside analysis of neutrophil phenotypes characterized by the differential expression of specific surface proteins (FcγRIII/CD16 and L-Selectin/CD62L) can be performed using automated point-of-care flow cytometry (5). Tissue damage leads to an influx of specific neutrophil phenotype subsets into the peripheral blood, such as neutrophils with an immature banded nucleus (“left shift”), which are characterized by low CD16 expression, as well as neutrophils with a hypersegmented nucleus, which are known for their regulatory functions (3, 5, 7). A visualization of neutrophil subsets allows the determination of the extent and kind of inflammation (8). However, the effect of major surgery on neutrophil phenotypes by automated flow cytometry has not been described previously.

Coronary artery bypass grafting (CABG) and pancreaticoduodenectomy (PPPD) are characterized by an extensive inflammatory response to surgery (9, 10). In CABG, this response is triggered by cardiopulmonary bypass, ischemia-reperfusion injury, and operative trauma. Inflammation occurring during pancreatic surgery is caused by an even more extensive operative trauma, malignancy, and neoadjuvant chemotherapy.

We hypothesize that neutrophil phenotypes in blood undergo changes after major surgery, depending on the type of surgery, due to differences in tissue damage-driven influx of damage-associated molecular patterns (DAMPs) and subsequent neutrophil subsets.

## 2 Methods

A prospective single-center cohort study was performed on adult patients undergoing major surgery, including pancreaticoduodenectomy (PPPD) or on-pump coronary artery bypass grafting (CABG). PPPD is a complex abdominal surgical procedure that involves removing parts of the pancreas, duodenum, and surrounding structures, often for cancer treatment, while CABG is a procedure that involves a bypass of stenotic coronary arteries to restore cardiac blood flow, characterized by the use of a heart-lung machine. This study was part of the “Biomarkers to guide perioperative management and improve outcome in high-risk surgery (BIGPROMISE)” study (11).

Blood samples were obtained after induction of anesthesia (preoperative sample) and directly after surgery. At each time point, 4 ml of blood was collected in a sodium-heparin tube (Greiner Bio-One GmbH, Kremsmünster, Austria) and analyzed within 30 min using an AQUIOS CL® “Load & Go” Flow Cytometer (Beckman Coulter Life Sciences, Miami, FL, USA). This fully automated flow cytometer is programmed to run a work-up and analysis protocol to examine marker proteins on the surface of neutrophils in the peripheral blood. All used antibodies were obtained from Beckman Coulter: CD16-FITC (clone 3G8), CD11b-PE (clone Bear1), CD62l-ECD (clone DREG56), CD10-PC5 (clone ALB1), and CD64-PC7 (clone 22). Raw flow cytometry files (.lmd) were exported from the flow cytometer and analyzed using Cytobank (Beckman Coulter; http://www.cytobank.org), a web-based flow cytometry analysis platform. Granulocytes were first identified by manually setting gates based on their forward scatter (FSC) and side scatter (SSC) properties. This gating strategy allows one to separate granulocytes, which are larger and more granular than other blood cells, from the rest of the cell population. Once the granulocytes were gated, mature neutrophils were identified within this population based on the expression of two surface markers, CD11b and CD16, while eosinophils were gated out due to their lack of CD16 expression (12) (Supplementary Figure S1). The heterogeneity of neutrophil phenotypes was evaluated through visual categorization. During acute inflammation, neutrophils can be divided into different subsets based on the expression of specific cell surface proteins (CD16/FcγRIII and CD62L/L-selectin) (13). In a recent study, we defined neutrophil phenotype categories (0–6) based on the occurrence of neutrophil subsets (Table 1) (6). Category 0 displays a normal, healthy, and homogenous neutrophil phenotype, while categories 1–6 show ordinal deviations. Category 1 shows a (larger) CD62L^low^ subset. Category 2 consists of a mixed subset in the lower left quadrant of the dot plot, where the expression of both CD16 and CD62L tends to be low. Category 3 consists of two small CD16^low^ and CD62L^low^ subsets that are fairly similar in size. Category 4 consists of larger CD16^low^ and CD62L^low^ subsets. Category 5 shows extensive CD16^low^ and CD62L^low^ subsets, with the CD16^low^ subset being bigger than the CD62L^low^ subset. Category 6 shows an immunophenotype of mainly neutrophil progenitors and CD62L^low^ neutrophils. In patients with trauma, injury severity was related to higher neutrophil phenotype categories 3–6. Neutrophil phenotype categories 0–6 were identified through visual assessment (5). This was blindly performed by two trained researchers based on the distribution of phenotypes that were identified according to their CD16 and CD62L expression.

**Table 1:** Distribution of perioperative neutrophil immunophenotype categories.

Immunophenotype category		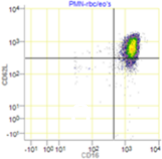 0	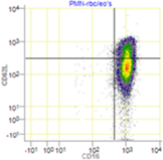 1	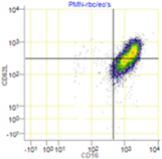 2	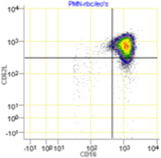 3	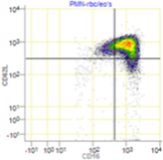 4	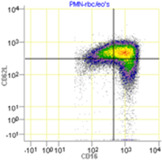 5	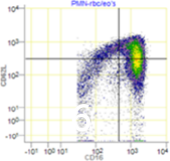 6
Cardiothoracic surgery	*N* = 24							
Preoperatively	*N* (%)	19 (79)	3 (13)	0	2 (8)	0	0	0
End of surgery	*N* (%)	0	0	0	0	17 (71)	7 (29)	0
Pancreatic surgery	*N* = 12							
Preoperatively	*N* (%)	10 (83)	2 (17)	0	0	0	0	0
End of surgery	*N* (%)	0	0	0	1 (8)	1 (8)	10 (83)	0
*P*–value								
Preoperatively		ns	ns	—	ns	—	—	—
End of surgery		—	—	—	ns	ns	0.007	—

*p*-values are depicted to show the distribution of patients between cardiothoracic surgery and pancreatic surgery either preoperatively or at the end of surgery.

All clinical parameters and endpoints were extracted from electronic medical files.

The chi-square test with Yates correction was used to investigate the association between surgery type and postoperative neutrophil phenotypes.

## 3 Results

We enrolled 36 patients, of whom 24 underwent CABG and 12 underwent pancreaticoduodenectomy. Patient characteristics are provided in Supplementary Table S1. The median age of the patients in the CABG group was 62 years [interquartile range (IQR) 58–64], and 20 (83%) patients were male. In the pancreatic surgery group, the median age was 68 years (IQR 62–75), and eight (67%) patients were male. Patients who underwent pancreatic surgery had lower American Society of Anesthesiologists (ASA) scores and longer hospital stays. None of the patients received preoperative steroids or immunosuppressive therapy.

**Figure 1:**
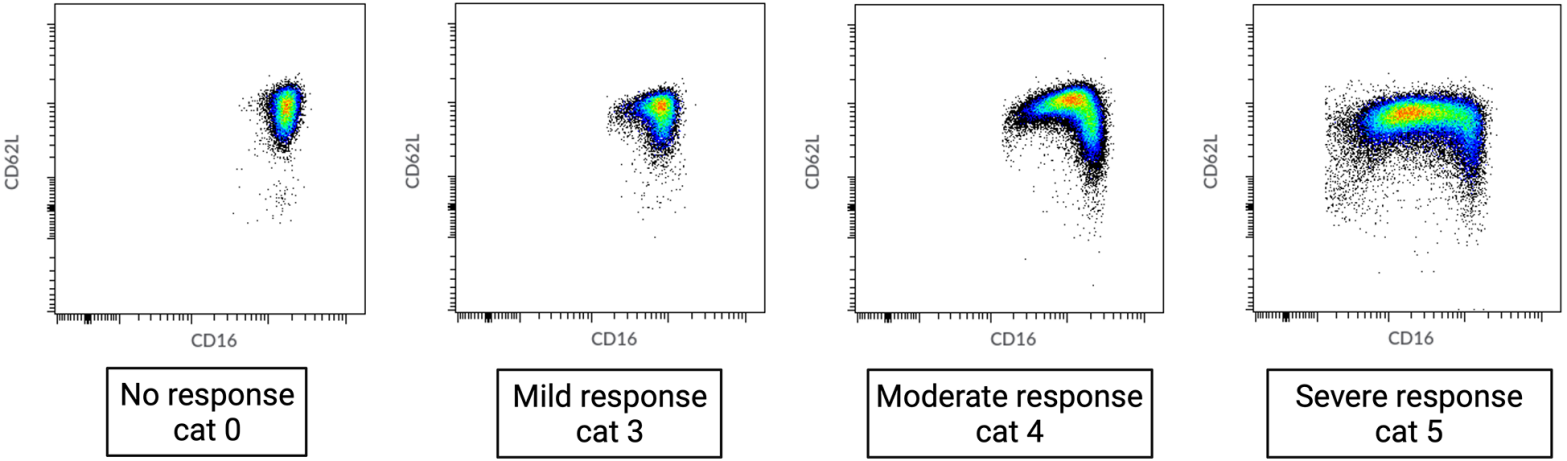
Range of neutrophil phenotypes occurring during systemic inflammation caused by different degrees of tissue damage. Cat, category as described in de Fraiture et al. (6).

Our results show a wide range of neutrophil responses, ranging from no response before major surgery (immune homeostasis) to the occurrence of extensive numbers of neutrophil subsets immediately after major surgery (Figure 1).

**Figure 2:**
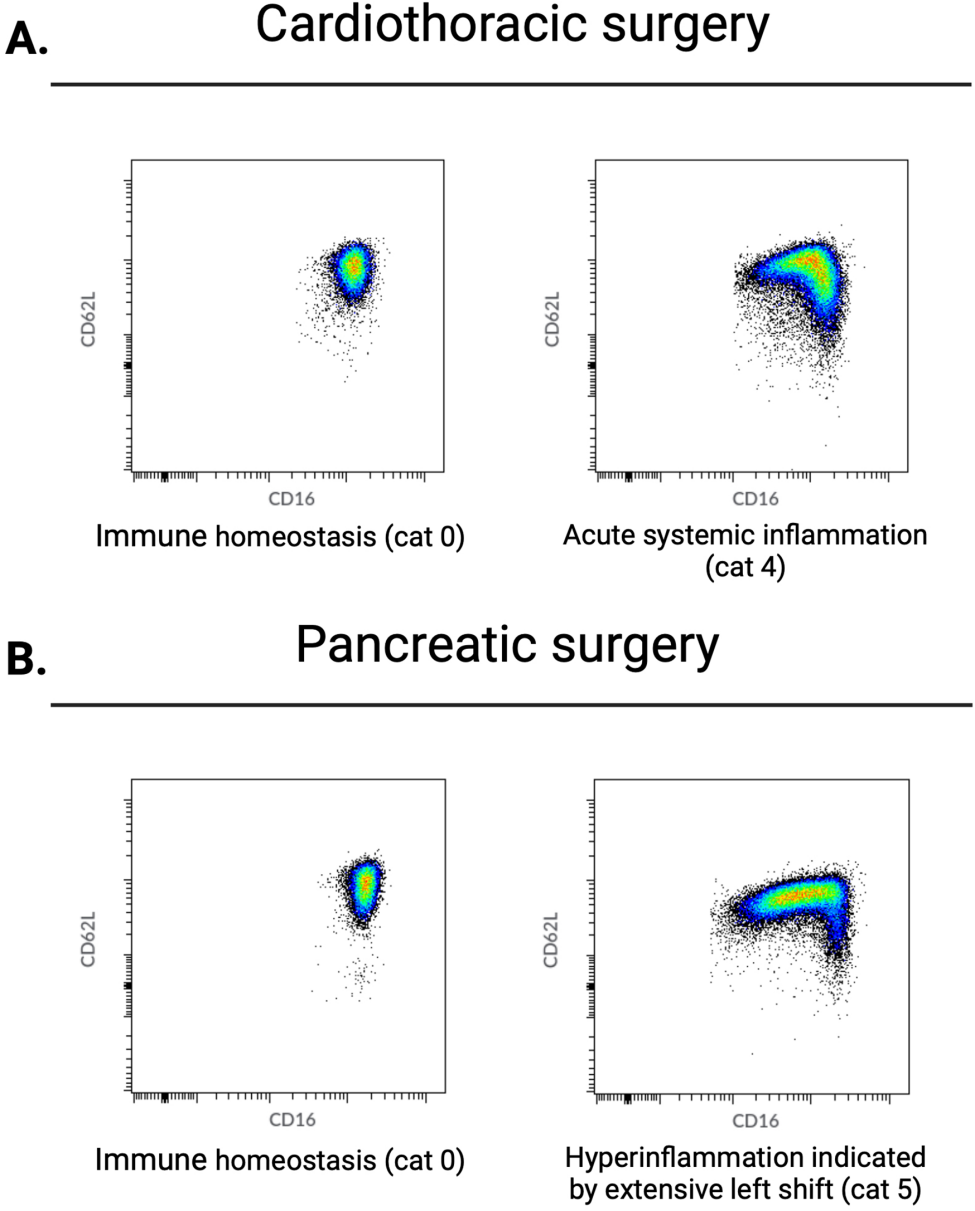
Perioperative data from patients who underwent either cardiothoracic or pancreatic surgery. Examples of neutrophil CD16/CD62L dot plots acquired by flow cytometry. From left to right: after anesthesia induction and directly postoperative. (**A**) Typical changes in neutrophil phenotypes observed in the majority of patients undergoing cardiothoracic surgery. Before surgery, no neutrophil subsets are present; directly after surgery, there is a left shift caused by an influx of immature CD16^low^ neutrophils. (**B**) Changes in neutrophil phenotypes observed directly after surgery in the majority of patients undergoing pancreatic surgery. Before surgery, there are no neutrophil subsets; directly after surgery, an extensive left shift caused by a larger influx of immature CD16^low^ neutrophils is observed. Cat, category as described in de Fraiture et al. (6).

Preoperatively, blood samples exhibited none or minimal neutrophil heterogeneity. All patients who underwent pancreatic surgery belonged to the physiologically normal neutrophil phenotype 0–1. In the CABG group, two (8%) patients showed modest preoperative systemic inflammation (category 3) (Table 1, Figure 2).

Postoperatively, all patients showed neutrophil subsets characteristic of acute systemic inflammation (category ≥3). With regard to the categories of neutrophil phenotypes, patients who underwent pancreatic surgery exhibited a more extensive inflammatory response than those who underwent CABG (Table 1, Figure 2). Specifically, 10 (83%) patients who underwent pancreatic surgery belonged to the neutrophil phenotype category 5 (representing hyperinflammation) while 7 (29%) belonged in the CABG group (*χ*^2^ = 7.37, *p* = 0.007).

## 4 Discussion

This study is the first to use fully automated flow cytometry to examine perioperative changes in neutrophil phenotypes after major surgery. It demonstrates heterogeneity in the severity of acute postoperative systemic inflammation, characterized by the occurrence of extensive numbers of neutrophil subsets immediately after major surgery. This is in line with prior findings in trauma patients, suggesting a DAMP-mediated response. Our results align with the concept of dysregulated postoperative inflammation in a subgroup of major surgery patients (2, 8). Specifically, our data indicate that the inflammatory response to pancreatic surgery is more pronounced than the response to CABG, which may reflect the different extents of tissue damage inflicted during these procedures.

In addition to quantifying the extent of tissue injury caused by surgical intervention, neutrophil phenotypes may help identify patients with postoperative dysregulation of the innate immune response. Hyperinflammation shortly after surgery could be part of a dysregulated systemic postoperative response that is often observed during and after major surgical interventions (1, 2). Such dysregulation that is characterized by both hyperinflammatory and immunosuppressive phases is often found during the occurrence of compensatory anti-inflammatory response syndrome (CARS) (14). This clinical feature is associated with immunosuppression, which makes patients more prone to infectious complications and is a risk factor for cancer recurrence (2). The ability to detect and monitor these immune states through neutrophil phenotyping may provide a more nuanced approach to postoperative patient management.

Earlier studies on postoperative inflammation have primarily focused on soluble inflammatory biomarkers such as C-reactive protein (CRP) and IL-6 (2). While these markers are known to increase in patients who develop inflammatory complications after abdominal surgery, they lack the specificity required to discern the source or nature of the inflammatory response (15–19). The neutrophil–lymphocyte ratio (NLR) offers a more direct measure of systemic inflammation, as elevated neutrophil counts during acute inflammation are reflected in a higher NLR. Postoperative increases in the NLR have been associated with poor outcomes in both cardiac and abdominal surgeries (20–22). Unlike the NLR, this study is the first to use automated flow cytometry for a more in-depth assessment of not only the quantification of overall neutrophil numbers but also the distinct characteristics of these cells. This phenotypic analysis provides deeper insights into the inflammatory response triggered by tissue damage, distinguishing the perioperative immune responses between pancreatic and cardiac surgeries in ways not previously described. Further studies are needed to validate the prognostic value of neutrophil subsets in predicting postoperative complications. By refining our understanding of postoperative inflammation through neutrophil phenotyping, we can identify patients at risk for immune dysregulation. As neutrophil subsets are measured immediately after surgery, early identification could guide targeted interventions, optimize perioperative care, and potentially reduce complications such as infections and delayed recovery.

In conclusion, this study demonstrates that activation of the systemic innate immune response, as quantified by changes in neutrophil phenotypes, is more pronounced following pancreaticoduodenectomy compared with CABG. The heterogeneity in neutrophil phenotypes observed after major surgery mirrors similar findings in trauma patients, indicating a common underlying mechanism (3, 5, 6, 8, 23). Future research should focus on validating these findings in larger cohorts and investigating the clinical implications of neutrophil phenotypes for predicting postoperative outcomes such as infectious complications. By refining our understanding of postoperative inflammation through neutrophil phenotyping, we may ultimately improve patient stratification and management in surgical settings.

## Data Availability

The raw data supporting the conclusions of this article will be made available by the authors upon reasonable request.

## Supplementary material

Supplementary Figure S1 Manual gating strategy for granulocytes based on forward and sideward scatter and for neutrophils based on containing CD11^high^/CD16^high^ cells.
