# Supplementary figures and images for: Neutrophil phenotypes quantify tissue damage caused by major surgery
Source: Front Surg. 2025 Mar 7;12:1494831. doi: 10.3389/fsurg.2025.1494831 (PMC11925952; doi:10.3389/fsurg.2025.1494831)

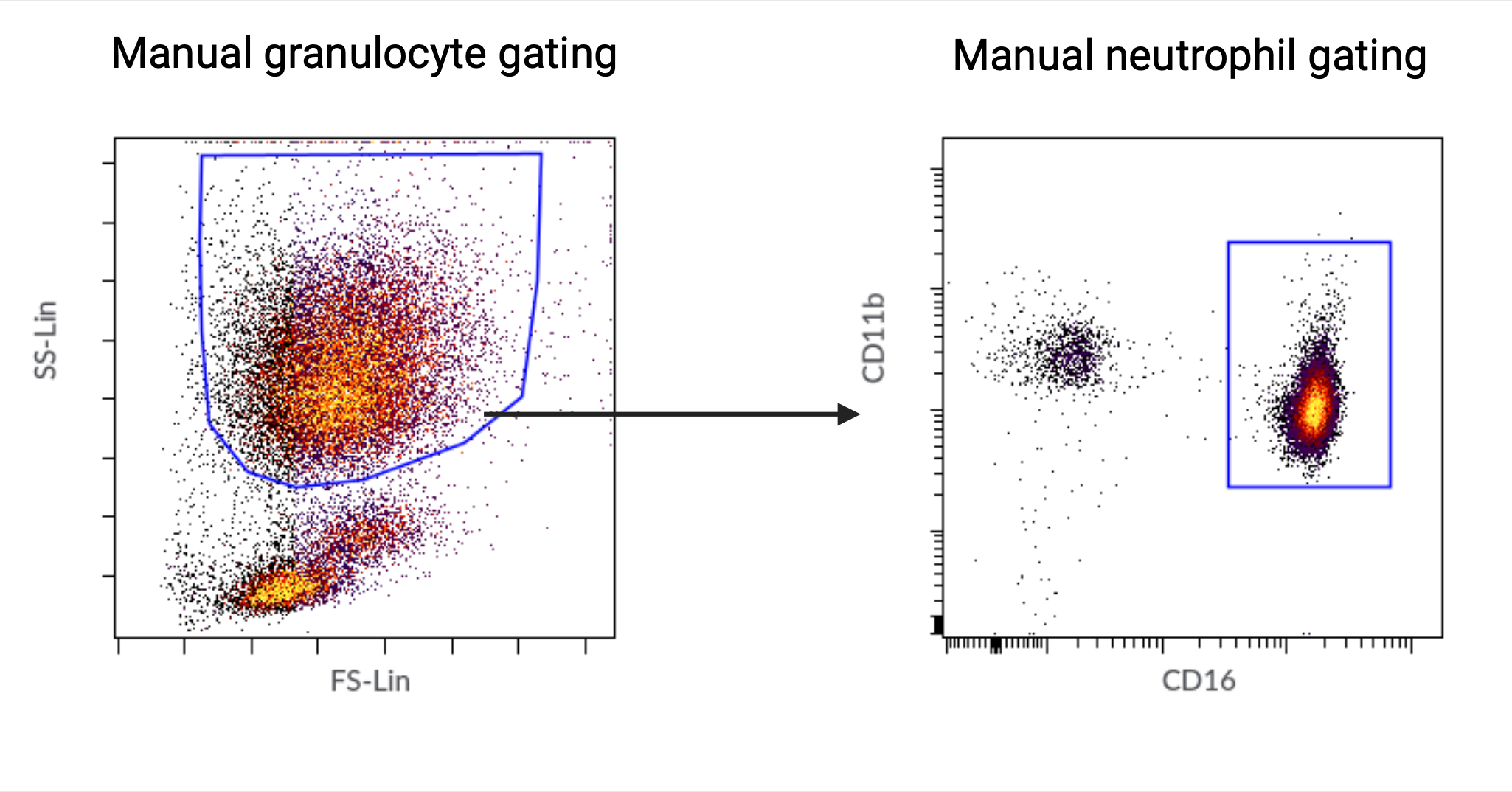

Supplement: Supplementary Figure S1 — Manual gating strategy for granulocytes based on forward and sideward scatter and for neutrophils based on containing CD11high/CD16high cells. [file Image1.jpeg]
