# Supplementary material for: Neutrophil phenotypes quantify tissue damage caused by major surgery
Source: Front Surg. 2025 Mar 7;12:1494831. doi: 10.3389/fsurg.2025.1494831 (PMC11925952; doi:10.3389/fsurg.2025.1494831)
Supplement: Supplementary file 2 [file Table1.docx]

Supplementary Table 1: Baseline characteristics and hospital/ICU length of stay of the study population, stratified by surgery type. BMI = body mass index, ASA = American Society of Anesthesiologists, ECOG = Eastern Cooperative Oncology Group, ICU = intensive care unit.

| **Variable** | **Cardiothoracic surgery** | **Pancreatic surgery** | **p value** |
| --- | --- | --- | --- |
| Age (median [IQR]) | 62.00 [57.75, 64.00] | 67.50 [62.25, 74.75] | 0.019 |
| Sex = Male (%) | 20 (83.3) | 8 (66.7) | 0.479 |
| BMI (median [IQR]) | 27.15 [26.00, 29.47] | 26.75 [23.88, 29.12] | 0.481 |
| Active smoker = Yes (%) | 2 (8.3) | 2 (16.7) | 0.851 |
| ASA class (%) |  |  | <0.001 |
| 2 | 0 (0.0) | 6 (50.0) |  |
| 3 | 18 (75.0) | 6 (50.0) |  |
| 4 | 6 (25.0) | 0 (0.0) |  |
| ECOG performance score |  |  | 0.536 |
| 0 | 14 (58.3) | 7 (58.3) |  |
| 1 | 8 (33.3) | 3 (25.0) |  |
| 2 | 2 (8.3) | 1 (8.3) |  |
| 3 | 0 (0.0) | 1 (8.3) |  |
| Preoperative steroids = Yes (%) | 0 (0.0) | 0 (0.0) |  |
| Preoperative immunosuppressive therapy = Yes (%) | 0 (0.0) | 0 (0.0) |  |
| Hospital length of stay (median [IQR]) | 5.50 [5.00, 7.25] | 12.50 [8.75, 16.75] | <0.001 |
| ICU length of stay (median [IQR]) | 1.00 [1.00, 1.00] | 1.00 [1.00, 1.50] | 0.576 |
